# Monkeypox: Oral manifestation as diagnostic indicator

**DOI:** 10.3205/dgkh000529

**Published:** 2024-12-16

**Authors:** Raquel D’Aquino Garcia Caminha, Gabriel de Toledo Telles-Araujo, Gabriel Araujo-Silva, Liliane Lins-Kusterer, Paulo Sérgio da Silva Santos

**Affiliations:** 1 Department of Surgery, Stomatology, Pathology and Radiology, Bauru Dental School, University of São Paulo, Bauru, Brazil; 2 School of Medicine, Federal University of Bahia, Salvador, Brazil; 3 Piracicaba Dental School, University of Campinas, Piracicaba, Brazil

**Keywords:** monkeypox, oral manifestation, diagnostic indicator

## Abstract

Lesions of monkeypox affect the oral mucosa in approximately 70% of infected patients and reported as the first clinical sign of the disease, manifesting as macules, papules, vesicles, or blisters, which are highly contagious and are followed by the appearance of lesions on the face and extremities of the body. These lesions have clinical aspects like recurrent herpes simplex, herpes zoster, and secondary syphilis and should be part of differential diagnoses.

The clinical course after initial oral manifestation is shown to support the clinical diagnosis.

## Abstract (de)

Läsionen an der Mundschleimhaut werden bei etwa 70% der infizierten Patienten beobachtet und als erstes klinisches Anzeichen der Krankheit angegeben. Sie äußern sich auch als Makula, Papeln, Bläschen oder Blasen, die hochgradig ansteckend sind und auf die Läsionen im Gesicht und an den Extremitäten folgen. Diese Läsionen weisen klinische Aspekte auf, die denen von rezidivierendem Herpes simplex, Herpes zoster und sekundärer Syphilis ähneln, weshalb Affenpocken zu den Differentialdiagnosen gehören sollten.

Als Hilfestellung für die klinische Diagnose wird der klinische Verlauf nach oraler Erstmanifestation dargestellt.

## Introduction

Monkeypox (MP) is a disease caused by the Monkeypox DNA virus (genus: Orthopoxvirus, family: Poxviridae), a zoonotic pathogen, which is spreading rapidly worldwide [1], [2]. Transmission of MP occurs through direct contact with saliva and respiratory secretions or from virus-infected lesions, through contact on contaminated surfaces, clothing, and objects, and through sexual intercourse [3], [4], [5], [6]. The presence of the virus is documented in semen [7], [8], [9], saliva [7], [9], nasopharyngeal swabs [7], [9], [10], urine [9], blood [7], [8], [9], [10], urine [7], [9], faeces [7], [9], rectal swab [9], skin lesions [8], [9] as well as in oral, pharyngeal and rectal lesions [9].

To date, the most affected population is homosexual patients, mainly males [11], aged between 21 and 40 years [2] and who have not been vaccinated against smallpox [1], [12], [13]. Penetration of the virus may occur via the oropharynx, nasopharynx, and/or skin and then a period of inoculation will occur with subsequent spread of the virus to the lymph nodes and an incubation period of approximately ranging from 7 to 21 days [14]. 

Clinically, the first signs and symptoms of the disease may appear from the 1^st^ to the 5^th^ day after contamination and manifest themselves through lymphadenopathies (prevalent characteristic), fever, fatigue, headache, and myalgias [15]. After the disappearance of fever, already in the second phase, the patient evolves with numerous rashes on the skin and mucous membranes such as the buccal, genital, cornea, and conjunctiva, with specific sequential stages of macules, papules, vesicles, pustules, crusts, in a typically centripetal manner and that after a few days or weeks heal, leaving the skin and mucosa integral [16], [17], [18], [19], [20]. The literature reports that some cases may also evolve with anal lesions, rectal pain, and penile edema [21], [22]. 

## Oral manifestation of monkeypox

**Figure 1:**
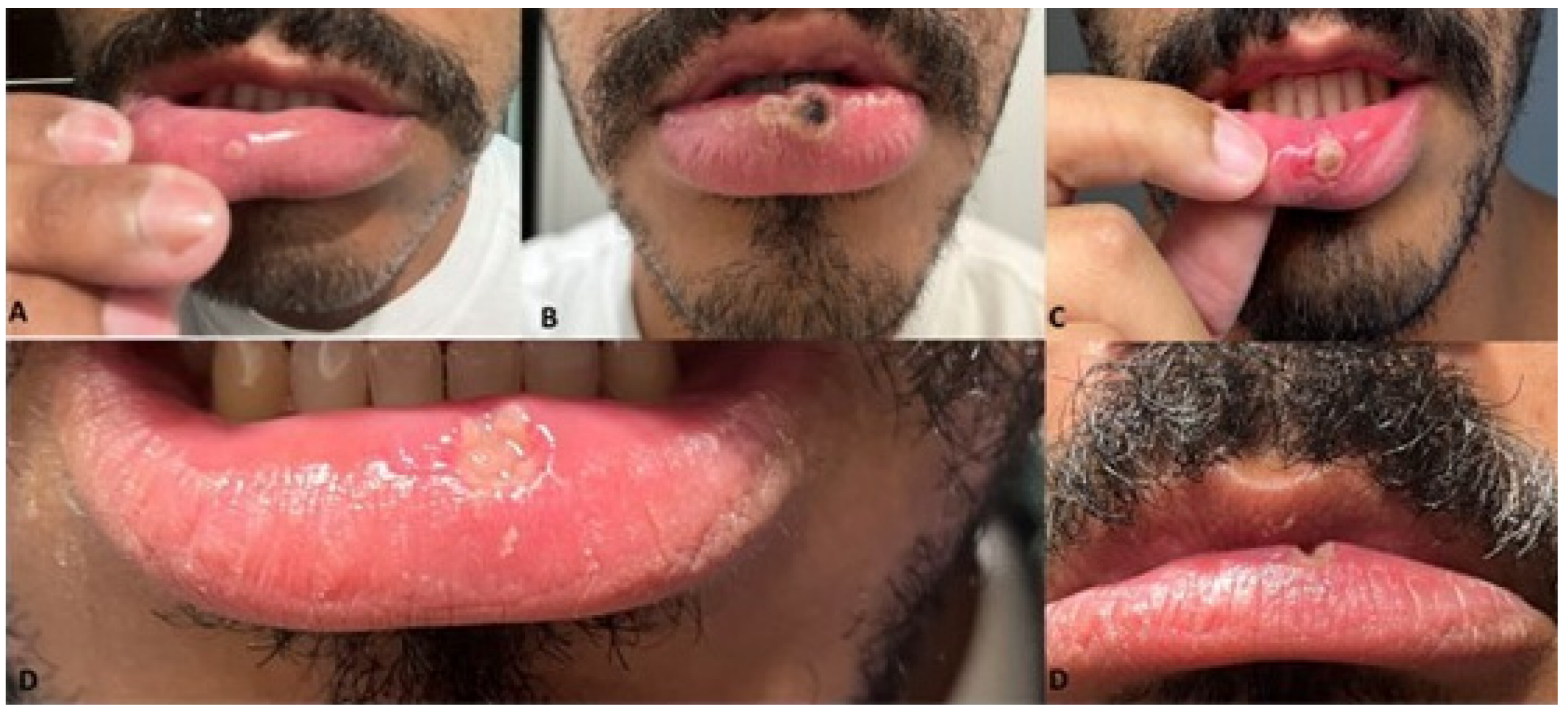
Clinical course of oral manifestation of monkeypox (A) Initial oral manifestation with the presence of a papule, (B) After 7 days a larger lesion with scaling and crusting, (C) After 12 days with an ulcer affecting the lip and lower lip mucosa, (D) After 15 days an ulcer on the lower lip with regression in size and the healing process.

Lesions affecting the oral mucosa have been observed in approximately 70% of infected patients [23] and are reported as the first clinical sign of the disease, also manifesting as macules, papules, vesicles, or blisters, which are highly contagious and are followed by the appearance of lesions on the face and extremities of the body. These lesions have clinical aspects similar to recurrent herpes, herpes zoster, and secondary syphilis and for this reason, MP should be part of the differential diagnoses [24]. In Figure 1 (Fig. 1) a typical clinical course is shown.

The oral lesions of MP present painful symptoms and interfere with feeding, generating a picture of dysphagia/odynophagia, dehydration, and malnutrition, worsening the systemic picture and quality of life [24]. It is noteworthy that oral lesions, regardless of the stage, are contaminated by the virus which consequently enables its spread [21].

## Discussion

The oral cavity may be one of the initial sites of the MP lesions, making it essential for health professionals to be informed about these signs and symptoms, which will allow early diagnosis, favoring the prognosis of the patient, besides minimizing infection to other people and the professional himself during clinical care [23]. The secretion from oral lesions can be collected with a swab to identify the DNA of the virus [25] and analyzed through the Polymerase Chain Reaction test for the diagnosis of MP, which is considered the gold standard for this diagnosis [23].

The clinical management of oral lesions of MP may include chemical control through mouthrinses with antimicrobials that will decrease the viral load in the oral cavity, such as 0.12% chlorhexidine without alcohol [26] and mechanical control through patient instruction on the best technique to perform oral hygiene, application of topical or systemic (acyclovir, fanciclovir, penciclovir, cidofovir) antivirals to the lesions [27]. In addition, it is also possible to associate the topical use of benzydamine hydrochloride, which will promote pain control through its anti-inflammatory, analgesic, and anesthetic action, providing greater comfort for the patient to eat properly [28], [29]. The use of low-power lasers presents great results to accelerate injury repair, analgesia, and anti-inflammatory effect, which may also be associated with photodynamic therapy to help reduce the viral load [30]. Laser therapy can also be applied in the management of MP, if its application is feasible, given the limited access to these patients due to the risk of spreading the disease. There are no studies in the literature that use these treatment modalities specifically for cases of MP, but there is scientific evidence of effective results in similar clinical situations, in the treatment of viral lesions, as mentioned above. 

The biosafety measures widely discussed during the Covid-19 pandemic [31], should be applied in suspected and/or confirmed cases of MP, such as a thorough anamnesis, use of N-95 or PFF-2 mask, face shield, disposable apron, goggles, abundant hand washing before and aftercare, in addition to all the recommended care with the care environment (including chair covered with disposable sheets) and contaminated materials [32], [33], [34], [35]. In outpatient care, it is recommended to guide the patient to come to the consultation wearing a mask, with the skin lesions covered, without a companion (who may be contaminated), and not bringing personal objects are also considered preventive measures [32]. 

During care, the patient should be instructed not to move around too much to minimize the chances of rupture of the skin lesions, which would increase the risk of spreading the virus present in these lesions [32]. 

## Conclusions

Oral lesions can be initial foci of MP, are symptomatic, and can be part of the early diagnosis of MP and its consequent treatment, improving the prognosis and quality of life of the affected patient. 

## Notes

### Competing interests

The authors declare that they have no competing interests.

### Authors’ ORCID

- Raquel D’Aquino Garcia Caminha:
https://orcid.org/0000-0002-8361-3894
- Gabriel de Toledo Telles-Araujo:
https://orcid.org/0000-0002-9577-2008
- Gabriel Araujo-Silva:
https://orcid.org/0000-0003-2235-9519
- Liliane Lins-Kusterer:
  https://orcid.org/0000-0003-3736-0002
- Paulo Sérgio da Silva Santos:
https://orcid.org/0000-0002-0674-3759

## References

[1] Kraemer MUG, Tegally H, Pigott DM, Dasgupta A, Sheldon J, Wilkinson E, Schultheiss M, Han A, Oglia M, Marks S, Kanner J, O'Brien K, Dandamudi S, Rader B, Sewalk K, Bento AI, Scarpino SV, de Oliveira T, Bogoch II, Katz R, Brownstein JS (2022). Tracking the 2022 monkeypox outbreak with epidemiological data in real-time. Lancet Infect Dis.

[2] Pan American Health Organization, World Health Organization (2022). Monkeypox Situation Report.

[3] Centers for disease control and prevention (2022). Monkeypox.

[4] Nolen LD, Osadebe L, Katomba J, Likofata J, Mukadi D, Monroe B, Doty J, Kalemba L, Malekani J, Kabamba J, Bomponda PL, Lokota JI, Balilo MP, Likafi T, Lushima RS, Tamfum JJ, Okitolonda EW, McCollum AM, Reynolds MG (2015). Introduction of Monkeypox into a Community and Household: Risk Factors and Zoonotic Reservoirs in the Democratic Republic of the Congo. Am J Trop Med Hyg.

[5] Nolen LD, Osadebe L, Katomba J, Likofata J, Mukadi D, Monroe B, Doty J, Hughes CM, Kabamba J, Malekani J, Bomponda PL, Lokota JI, Balilo MP, Likafi T, Lushima RS, Ilunga BK, Nkawa F, Pukuta E, Karhemere S, Tamfum JJ, Nguete B, Wemakoy EO, McCollum AM, Reynolds MG (2016). Extended Human-to-Human Transmission during a Monkeypox Outbreak in the Democratic Republic of the Congo. Emerg Infect Dis.

[6] McCollum AM, Damon IK (2014). Human monkeypox. Clin Infect Dis.

[7] Antinori A, Mazzotta V, Vita S, Carletti F, Tacconi D, Lapini LE, D'Abramo A, Cicalini S, Lapa D, Pittalis S, Puro V, Rivano Capparuccia M, Giombini E, Gruber CEM, Garbuglia AR, Marani A, Vairo F, Girardi E, Vaia F, Nicastri E, INMI Monkeypox Group (2022). Epidemiological, clinical and virological characteristics of four cases of monkeypox support transmission through sexual contact, Italy, May 2022. Euro Surveill.

[8] Noe S, Zange S, Seilmaier M, Antwerpen MH, Fenzl T, Schneider J, Spinner CD, Bugert JJ, Wendtner CM, Wölfel R (2023). Clinical and virological features of first human monkeypox cases in Germany. Infection.

[9] Peiró-Mestres A, Fuertes I, Camprubí-Ferrer D, Marcos MÁ, Vilella A, Navarro M, Rodriguez-Elena L, Riera J, Català A, Martínez MJ, Blanco JL, Hospital Clinic de Barcelona Monkeypox Study Group (2022). Frequent detection of monkeypox virus DNA in saliva, semen, and other clinical samples from 12 patients, Barcelona, Spain, May to June 2022. Euro Surveill.

[10] Adler H, Gould S, Hine P, Snell LB, Wong W, Houlihan CF, Osborne JC, Rampling T, Beadsworth MB, Duncan CJ, Dunning J, Fletcher TE, Hunter ER, Jacobs M, Khoo SH, Newsholme W, Porter D, Porter RJ, Ratcliffe L, Schmid ML, Semple MG, Tunbridge AJ, Wingfield T, Price NM, NHS England High Consequence Infectious Diseases (Airborne) Network (2022). Clinical features and management of human monkeypox: a retrospective observational study in the UK. Lancet Infect Dis.

[11] Mahase E (2022). Seven monkeypox cases are confirmed in England. BMJ.

[12] Otu A, Ebenso B, Walley J, Barceló JM, Ochu CL (2022). Global human monkeypox outbreak: atypical presentation demanding urgent public health action. Lancet Microbe.

[13] Kozlov M (2022). Monkeypox outbreaks: 4 key questions researchers have. Nature.

[14] Hahon N, McGavran MH (1961). Air-borne infectivity of the variola-vaccinia group of poxviruses for the cynomolgus monkey, Macaca irus. J Infect Dis.

[15] Patel A, Bilinska J, Tam JCH, Da Silva Fontoura D, Mason CY, Daunt A, Snell LB, Murphy J, Potter J, Tuudah C, Sundramoorthi R, Abeywickrema M, Pley C, Naidu V, Nebbia G, Aarons E, Botgros A, Douthwaite ST, van Nispen Tot Pannerden C, Winslow H, Brown A, Chilton D, Nori A (2022). Clinical features and novel presentations of human monkeypox in a central London centre during the 2022 outbreak: descriptive case series. BMJ.

[16] Adler H, Gould S, Hine P, Snell LB, Wong W, Houlihan CF, Osborne JC, Rampling T, Beadsworth MB, Duncan CJ, Dunning J, Fletcher TE, Hunter ER, Jacobs M, Khoo SH, Newsholme W, Porter D, Porter RJ, Ratcliffe L, Schmid ML, Semple MG, Tunbridge AJ, Wingfield T, Price NM, NHS England High Consequence Infectious Diseases (Airborne) Network (2022). Clinical features and management of human monkeypox: a retrospective observational study in the UK. Lancet Infect Dis.

[17] Minhaj FS, Ogale YP, Whitehill F, Schultz J, Foote M, Davidson W, Hughes CM, Wilkins K, Bachmann L, Chatelain R, Donnelly MAP, Mendoza R, Downes BL, Roskosky M, Barnes M, Gallagher GR, Basgoz N, Ruiz V, Kyaw NTT, Feldpausch A, Valderrama A, Alvarado-Ramy F, Dowell CH, Chow CC, Li Y, Quilter L, Brooks J, Daskalakis DC, McClung RP, Petersen BW, Damon I, Hutson C, McQuiston J, Rao AK, Belay E, McCollum AM, Monkeypox Response Team 2022 (764-9). Monkeypox Outbreak - Nine States, May 2022.

[18] Ogoina D, Iroezindu M, James HI, Oladokun R, Yinka-Ogunleye A, Wakama P, Otike-Odibi B, Usman LM, Obazee E, Aruna O, Ihekweazu C (2020). Clinical Course and Outcome of Human Monkeypox in Nigeria. Clin Infect Dis.

[19] Mahase E (2022). Monkeypox: What do we know about the outbreaks in Europe and North America? BMJ.

[20] Brown K, Leggat PA (2016). Human Monkeypox: Current State of Knowledge and Implications for the Future. Trop Med Infect Dis.

[21] Patel A, Bilinska J, Tam JCH, Da Silva Fontoura D, Mason CY, Daunt A, Snell LB, Murphy J, Potter J, Tuudah C, Sundramoorthi R, Abeywickrema M, Pley C, Naidu V, Nebbia G, Aarons E, Botgros A, Douthwaite ST, van Nispen Tot Pannerden C, Winslow H, Brown A, Chilton D, Nori A (2022). Clinical features and novel presentations of human monkeypox in a central London centre during the 2022 outbreak: descriptive case series. BMJ.

[22] Thornhill JP, Barkati S, Walmsley S, Rockstroh J, Antinori A, Harrison LB, Palich R, Nori A, Reeves I, Habibi MS, Apea V, Boesecke C, Vandekerckhove L, Yakubovsky M, Sendagorta E, Blanco JL, Florence E, Moschese D, Maltez FM, Goorhuis A, Pourcher V, Migaud P, Noe S, Pintado C, Maggi F, Hansen AE, Hoffmann C, Lezama JI, Mussini C, Cattelan A, Makofane K, Tan D, Nozza S, Nemeth J, Klein MB, Orkin CM, SHARE-net Clinical Group (2022). Monkeypox Virus Infection in Humans across 16 Countries - April-June 2022. N Engl J Med.

[23] Samaranayake L, Anil S (2022). The Monkeypox Outbreak and Implications for Dental Practice. Int Dent J.

[24] World Health Organization (2022). Clinical management and infection prevention and control for monkeypox: interim rapid response guidance, 10 June 2022.

[25] Li Y, Olson VA, Laue T, Laker MT, Damon IK (2006). Detection of monkeypox virus with real-time PCR assays. J Clin Virol.

[26] Santos DSF, Peralta-Mamani M, Brandão FS, Andrade FB, Cruvinel T, Santos PSDS (2022). Could polyhexanide and chlorine dioxide be used as an alternative to chlorhexidine? A systematic review. Sao Paulo Med J.

[27] Hirata CH (2015). Oral manifestations in AIDS. Braz J Otorhinolaryngol.

[28] Epstein JB, Silverman S, Paggiarino DA, Crockett S, Schubert MM, Senzer NN, Lockhart PB, Gallagher MJ, Peterson DE, Leveque FG (2001). Benzydamine HCl for prophylaxis of radiation-induced oral mucositis: results from a multicenter, randomized, double-blind, placebo-controlled clinical trial. Cancer.

[29] Chitapanarux I, Tungkasamit T, Petsuksiri J, Kannarunimit D, Katanyoo K, Chakkabat C, Setakornnukul J, Wongsrita S, Jirawatwarakul N, Lertbusayanukul C, Sripan P, Traisathit P (2018). Randomized control trial of benzydamine HCl versus sodium bicarbonate for prophylaxis of concurrent chemoradiation-induced oral mucositis. Support Care Cancer.

[30] Ajmal M (2021). Effectiveness of photodynamic therapy as an adjunct to topical antiviral therapy in the treatment of herpes labialis: A randomized controlled clinical trial. Photodiagnosis Photodyn Ther.

[31] de Toledo Telles-Araujo G, Caminha RDG, Kallás MS, Sipahi AM, da Silva Santos PS (2020). Potential mouth rinses and nasal sprays that reduce SARS-CoV-2 viral load: What we know so far? Clinics (Sao Paulo).

[32] UK Health Security Agency (UKHSA), Public Health Scotland, Public Health Wales, Public Health Agency Northern Ireland (2022). Guidance – Principles for control of non-HCID mpox in the UK: 4 nations consensus statement.

[33] Samaranayake L (2018). Essential microbiology for dentistry.

[34] Tsagkaris C, Eleftheriades A, Laubscher L, Vladyckuk V, Papadakis M (2023). Viruses monkeying around with surgical safety: Monkeypox preparedness in surgical settings. J Med Virol.

[35] Cunha BE (2004). Monkeypox in the United States: an occupational health look at the first cases. AAOHN J.

[36] 24 ([Accessed). Ministério da Saúde.

